# Transmission of the Bean-Associated Cytorhabdovirus by the Whitefly *Bemisia tabaci* MEAM1

**DOI:** 10.3390/v12091028

**Published:** 2020-09-15

**Authors:** Bruna Pinheiro-Lima, Rita C. Pereira-Carvalho, Dione M. T. Alves-Freitas, Elliot W. Kitajima, Andreza H. Vidal, Cristiano Lacorte, Marcio T. Godinho, Rafaela S. Fontenele, Josias C. Faria, Emanuel F. M. Abreu, Arvind Varsani, Simone G. Ribeiro, Fernando L. Melo

**Affiliations:** 1 Embrapa Recursos Genéticos e Biotecnologia, Brasília DF 70770-017, Brazil; pinheiro.limab@gmail.com (B.P.-L.); dionebio@gmail.com (D.M.T.A.-F.); andrezactg@hotmail.com (A.H.V.); cristiano.lacorte@embrapa.br (C.L.); marcioslash@gmail.com (M.T.G.); emanuel.abreu@embrapa.br (E.F.M.A.); 2 Departamento de Fitopatologia, Instituto de Biologia, Universidade de Brasília, Brasília DF 70275-970, Brazil; rcpcarvalho@unb.br; 3 Departamento de Biologia Celular, Instituto de Biologia, Universidade de Brasília, Brasília DF 70275-970, Brazil; 4 Departamento de Fitopatologia, Escola Superior de Agricultura Luiz de Queiroz, Piracicaba SP 13418-900, Brazil; ewkitaji@usp.br; 5 The Biodesign Center for Fundamental and Applied Microbiomics, Center for Evolution and Medicine School of Life Sciences, Arizona State University, Tempe, AZ 85287-5001, USA; rafasfontenele@gmail.com (R.S.F.); avarsani@gmail.com (A.V.); 6 Embrapa Arroz e Feijão, Goiânia GO 75375-000, Brazil; josias.faria@embrapa.br; 7 Structural Biology Research Unit, Department of Integrative Biomedical Sciences, University of Cape Town, Observatory, Cape Town 7701, South Africa

**Keywords:** common bean, *Phaseolus vulgaris*, cytorhabdovirus, whitefly, *Bemisia tabaci*, vector, virus transmission, virus evolution

## Abstract

The knowledge of genomic data of new plant viruses is increasing exponentially; however, some aspects of their biology, such as vectors and host range, remain mostly unknown. This information is crucial for the understanding of virus–plant interactions, control strategies, and mechanisms to prevent outbreaks. Typically, rhabdoviruses infect monocot and dicot plants and are vectored in nature by hemipteran sap-sucking insects, including aphids, leafhoppers, and planthoppers. However, several strains of a potentially whitefly-transmitted virus, papaya cytorhabdovirus, were recently described: (i) bean-associated cytorhabdovirus (BaCV) in Brazil, (ii) papaya virus E (PpVE) in Ecuador, and (iii) citrus-associated rhabdovirus (CiaRV) in China. Here, we examine the potential of the *Bemisia tabaci* Middle East-Asia Minor 1 (MEAM1) to transmit BaCV, its morphological and cytopathological characteristics, and assess the incidence of BaCV across bean producing areas in Brazil. Our results show that BaCV is efficiently transmitted, in experimental conditions, by *B. tabaci* MEAM1 to bean cultivars, and with lower efficiency to cowpea and soybean. Moreover, we detected BaCV RNA in viruliferous whiteflies but we were unable to visualize viral particles or viroplasm in the whitefly tissues. BaCV could not be singly isolated for pathogenicity tests, identification of the induced symptoms, and the transmission assay. BaCV was detected in five out of the seven states in Brazil included in our study, suggesting that it is widely distributed throughout bean producing areas in the country. This is the first report of a whitefly-transmitted rhabdovirus.

## 1. Introduction

Rhabdoviruses (family *Rhabdoviridae*) are a group of negative-sense, single-stranded RNA viruses that infect plants, vertebrate animals, and invertebrate animals. They cause harmful diseases in humans and animals and can cause high yield losses in crops. Plant-infecting rhabdoviruses are currently taxonomically assigned to six genera [1]. Members of the *Dichorhavirus* genus have a bi-segmented genome, infect di- and monocotyledonous plants, and are transmitted by *Brevipalpus* mites. Viruses belonging to the *Varicosavirus* genus also have a bi-segmented genome, infect plants of the Compositae and Solanaceae families, and are transmitted by zoospores of the fungus *Olpidium brassicae*. Non-segmented plant rhabdoviruses infect mono- and dicot plants and are vectored in nature by hemipteran sap-sucking insects, including aphids, leafhoppers, and planthoppers [2]. Moreover, there is a close relationship between plant rhabdoviruses and their vectors, and each virus may be vectored by one species or a few closely related ones [3]. Viruses that replicate within the nuclei of infected plant cells are assigned to the genera *Alphanucleorhabdovirus*, *Betanucleorhabdovirus*, and *Gammanucleorhabdovirus,* while those that multiply in the cell cytoplasm belong to the *Cytorhabdovirus* [2,4]. Insect and mite-transmitted rhabdoviruses also replicate in their arthropod vectors and are transmitted in a persistent propagative manner [3,5]. However, no information on vectors and transmission characteristics is available for most of these viruses [5].

Bean-associated cytorhabdovirus (BaCV) was identified in transgenic bean golden mosaic virus (BGMV)-resistant common bean lines [6]. The BaCV genome has a 3′-N-P-M-G-L-5′ [nucleocapsidprotein (N), phosphoprotein (P), matrix protein (M), glycoprotein (G), and RNA-dependent RNA polymerase (RdRp) protein (L)] organization that is typical of rhabdoviruses and between P and M, the BaCV genome encodes two accessory genes, P3 and P4 [6]. A closely related virus with a genome sequence identity of 97% named papaya virus E (PpVE) has been reported from papaya plants in Ecuador [7]. Based on the high sequence identity between the two virus sequences, it was proposed that the virus species would be named *Papaya cytorhabdovirus* with strains PpVE infecting papayas and BaCV infecting beans [8,9].

To characterize the bean-infecting cytorhabdovirus strain in detail, we carried out the molecular cloning and defined its morphological and cytopathological characteristics. Moreover, we observed a high prevalence of BaCV in bean fields in Brazil and determined the efficient transmission of BaCV by the whitefly *Bemisia tabaci* Middle East-Asia Minor 1 (MEAM1), a hitherto unknown feature for a plant rhabdovirus.

## 2. Materials and Methods

### 2.1. Plant Material

**Figure 1:**
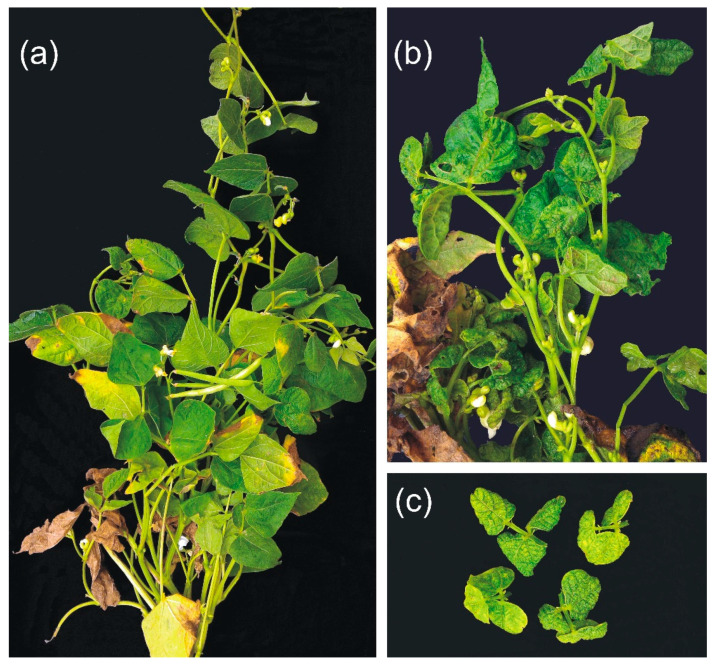
Symptoms in common bean plants collected in a commercial field in Luziânia, Goiás state with mixed infection by bean-associated cytorhabdovirus (BaCV), cowpea mild mottle virus (CPMMV), and bean golden mosaic virus (BGMV) and whitefly colonization. (**a**) Mosaic and leaf wrinkling. (**b**) Mosaic, severe leaf distortion, and deformation. (**c**) Detail of leaves with reduced area, yellow mosaic, and severe crinkling and curling.

Fifteen common bean plants (cultivar ‘Pérola’) with typical virus symptoms of mosaic, leaf distortion, crumpling, and dwarfing (Figure 1) were collected in a commercial field in Luziânia, Goiás State, in June 2016. Leaf samples from the 15 plants were detached and stored at −80 °C. Six plants were transplanted to soil-filled pots and maintained in a screen-protected cage, without removing the abundant whitefly colonies present in the plant leaves. One of the transplanted plants survived and was used for virus transmission and cloning of the genome of BaCV-Luz.

### 2.2. Distribution of BaCV in Common Beans in Brazil

**Figure 2:**
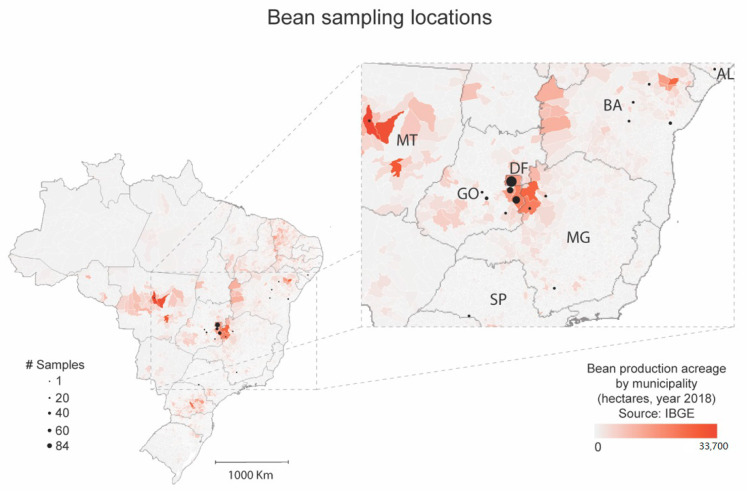
Summary of the distribution of the bean sampling locations at Alagoas (AL), Bahia (BA), Goiás (GO), Minas Gerais (MG), Mato Grosso (MT), São Paulo (SP) states and Distrito Federal (DF). Each municipality was colored in red according to the bean planted area (in hectare), obtained from the Brazilian Institute of Geography (https://www.ibge.gov.br/) [10]. The black circles represent the sample sites, and their size is proportional to the number of collected plants. The samples were collected between 2007 and 2018.

Initially, the 15 plants collected in Luziânia were examined by RT-PCR or PCR for the presence of bean-infecting viruses frequently found in Brazil, the whitefly-transmitted CPMMV (family *Betaflexiviridae*; genus *Carlavirus*), BGMV, and macroptilium yellow spot virus—MaYSV (family *Geminiviridae*; genus *Begomovirus*), the chrysomelid beetle-transmitted bean rugose mosaic virus—BRMV (family *Secoviridae*; genus *Comovirus*), and the recently identified cytorhabdovirus BaCV. To determine the occurrence of BaCV in different areas in Brazil (Figure 2), additional bean samples were collected from experimental or commercial bean fields between 2016 to 2018, including Brasília (*n* = 30), Distrito Federal—DF; Santo Antônio de Goiás (*n* = 26), Luziânia (*n* = 15), Cristalina (*n* = 43), Urutai (*n* = 1), and Araçu (*n* = 1) in Goiás State—GO; Sorriso, Mato Grosso State—MT (*n* = 2); Bonfinópolis de Minas (*n* = 3), Paracatu (*n* = 1), Três Pontas (*n* = 5) in Minas Gerais State; and Arapiraca (*n* = 5) in Alagoas State. In addition, we analyzed bean samples from our archived collection. Plants collected in Brasília, DF in 2007 (*n* = 2), PAD/DF Paranoá, DF in 2012 (*n* = 11), and Riacho Fundo, DF in 2015 (*n* = 41); Cruz das Almas (*n* = 8), Morro do Chapéu (*n* = 4), Piritiba (*n* = 3), and Antônio Gonçalves (*n* = 2) in Bahia State in 2015; and Palmital (*n* = 1) in São Paulo State in 2015 were screened for BaCV infection. In total, 219 bean plants were screened for the presence of BaCV, CPMMV, and BGMV (41 BGMV-immune cultivar ‘BRS FC 401 RMD’ plants were not tested for BGMV).

### 2.3. RNA and DNA Extraction

Total RNA was extracted from ~100 mg of plant leaf tissue (pulverized in liquid nitrogen) using the TRIzol Reagent (Invitrogen, Carlsbad, CA, USA) according to the manufacturer’s instructions. Total RNA was also extracted from (i) a group of 30 whitefly individuals, (ii) from one single individual, and (iii) parts of one single whitefly (head/thorax or abdomen). One fourth and one-tenth of the reagent’s volumes of the standard TRIzol Reagent protocol were used for 30 whiteflies and one or parts of an insect, respectively. Total DNA was extracted from leaves and whiteflies using the CTAB protocol [11].

### 2.4. RT-PCR, PCR, and Cloning

The sequences and characteristics of all primers used in this study are summarized in Table S1. To determine the complete genome sequence of BaCV-Luz, first we determined the 5′ and 3′ ends by rapid amplification of cDNA ends (RACE) as previously described [6,12,13]. Next, based on the genome sequence of BaCV-GO [6] and BaCV-Luz leader and trailer sequences, primers were designed to amplify the virus genome by RT-PCR in six fragments overlapping in at least 150 nt. The cDNA was synthesized with 5 μL of total RNA (approximately 1 μg) using SuperScript III Reverse Transcriptase (Invitrogen, Carlsbad, CA, USA), Anchored Oligo(dT)20 (Invitrogen, Carlsbad, CA, USA) and random primers [14]. One microliter of cDNA was used in PCR reactions with LongAmp Taq DNA Polymerase (New England Biolabs, Ipswich, MA, USA) and specific primers for each fragment (Table S1). Amplicons were gel-purified, cloned into PCR 2.1 TOPO TA vector (Invitrogen, Carlsbad, CA, USA) and sequenced. At least two clones of each fragment were sequenced at Macrogen Inc. (Seoul, Korea).

Detection of BaCV (in plants and whiteflies), CPMMV (in plants and whiteflies), and BRMV (in plants) was carried out by RT-PCR using the cDNA prepared as described above as template. PCR reactions were performed with Taq DNA Polymerase (Invitrogen, Carlsbad, CA, USA) using 1 μL of cDNA and primers BaCV_1F/BaCV_1579R, CPMMV_4000F/CPMMV_4500R [15], and BRMV1_76F/BRMV1_521R [6] specific for BaCV, CPMMV, and BRMV, respectively. For BGMV and MaYSV PCR-based infection surveys of field samples, total extracted plant DNA was used as template and primers BGMV_HPXHO/BGMV_HPKPN for BGMV [16] and MaYSV-249F/MaYSV-1083R for MaYSV. A portion of the PCR amplicons was verified by cloning in PCR 2.1 TOPO TA vector (Invitrogen, Carlsbad, CA, USA) and Sanger sequencing.

Whiteflies populations from the field, as well as from the Universidade de Brasília rearing facility, were identified as *B. tabaci* MEAM1 by genotyping the insects using PCR-RFLP as described by Bosco, et al. [17]. Amplification of a region of the mitochondrial cytochrome oxidase I gene (mtCOI) was undertaken by PCR using whitefly total DNA as template and the primers COI-Fw/COI-Rv [17] (Table S1). The amplicons were digested with Taq I endonuclease (New England Biolabs, Ipswich, MA, USA). The digestion products were resolved in a 1.2% agarose gel electrophoresis, and the profile of the bands was compared with the profiles for *B. tabaci* MEAM1 (synonym biotype B) and *B. tabaci* MED (synonym biotype Q) [17].

### 2.5. Sequence Analysis

All sequences generated in this study were trimmed and assembled using Geneious software (v. 11, Biomatters, Auckland, New Zealand) [18]. The sequence identity was confirmed by BLASTn analysis [19]. The complete genome of BaCV-Luz isolate was deposited in GenBank under the accession number MT811775. The RdRp amino acid sequence of BaCV-Luz was aligned with those of all cytorhabdoviruses available in GenBank (as by May 2020) (Table S2) using MAFFT algorithm [20] implemented in Geneious. Maximum likelihood (ML) phylogenetic tree was inferred using IQ-TREE [21], with node support estimated with the Shimodaira–Hasegawa approximate likelihood ratio test (SH-aLRT) [22]. Moreover, the amino acid sequences of the glycoproteins encoded by the cytorhabdoviruses were used to generate a sequence similarity network using the Enzyme Function Initiative–Enzyme Similarity Tool (EFI–EST) [23] with an alignment score threshold of 35 and minimum E-value threshold of 1 × 10^−5^. The network was visualized in Cytoscape v3.7.1 [24]. Pairwise sequence identity comparisons were performed using the Sequence Demarcation Tool (SDT) v.1.2 [25].

### 2.6. BaCV Transmission by B. tabaci MEAM1

**Figure 3:**
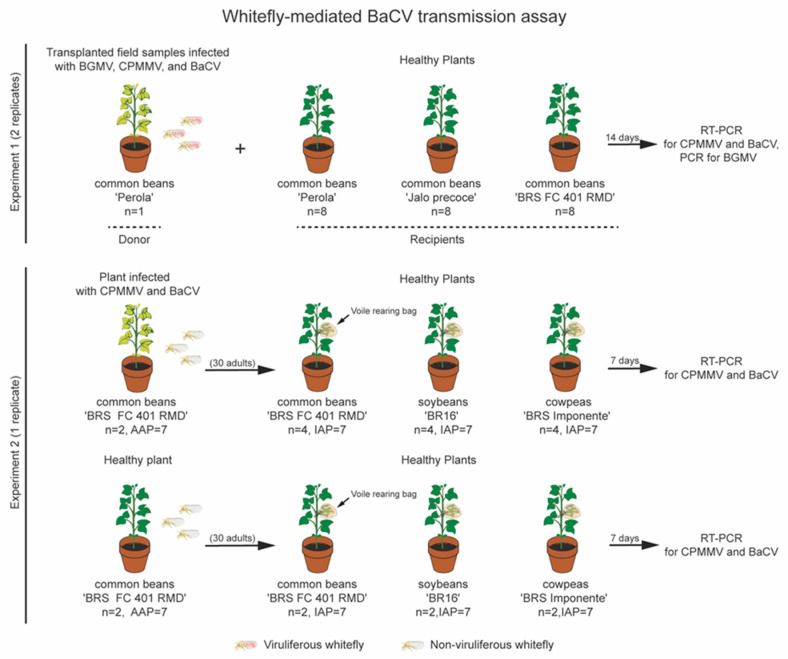
Whitefly (*Bemisia tabaci* Middle East-Asia Minor 1 (MEAM1)) mediated transmission of BaCV. Outline of experimental procedures: In experiment 1, a common bean plant with whitefly colonies from the field was used as an inoculum source for BaCV (BGMV and CPMMV) transmission to bean plants ‘Jalo’, ‘Pérola’ and ‘BRS FC 401 RMD’. After 14 days, samples were collected, and BaCV (CPMMV and BGMV) transmission confirmed by RT-PCR and PCR. In experiment 2, whiteflies were exposed to BaCV- (and CPMMV)-infected plants for a 7-days acquisition period. Thirty whiteflies were transferred from the inoculum source plant to a healthy plant leaf wrapped by a voile rearing bag for a 7-days inoculation period. After seven days, samples were collected and tested by RT-PCR. The same procedure was applied to healthy plants with aviruliferous whiteflies as controls.

After confirming the infection by BaCV, CPMMV, and BGMV, a bean plant colonized by whiteflies transplanted from the field was used as an inoculum source for transmission tests (Figure 3). Initially, eight young bean seedlings each of cultivars ‘Pérola’, ‘Jalo’, and ‘BRS FC 401 RMD’ were placed in the cage with the whitefly-infested plant. Seven days later, another set of eight seedlings of each cultivar was placed inside the same cage. Plant samples were collected 14 days after their introduction into the cage and tested for the presence of BaCV, CPMMV, and BGMV. Since ‘BRS FC 401 RMD’ plants are immune to BGMV [26], they were not tested for BGMV infection. Whiteflies were also collected and tested for the presence of BaCV. Following this, the BaCV-Luz isolate was maintained in ‘BRS FC 401 RMD’ plants by introducing plantlets every three to four weeks in the cage, and when the population of whiteflies was declining, adult individuals from the rearing facility from the Universidade de Brasília were added to the cage for BaCV-Luz isolate maintenance until 2018.

To further refine the transmission tests, two three-week old ‘BRS FC 401 RMD’ plants infected with BaCV-Luz (and CPMMV) were removed from the cage, sprayed with imidacloprid (32 mg/L) to eliminate any whiteflies present in the plants, transferred to a bugdorm tent, and kept in a greenhouse at room temperature for 10 days. *B. tabaci* MEAM1 individuals were reared in cabbage plants (*Brassica oleraceae* var. *capitata* L.) at the Biological Experimental Station of the Universidade de Brasília. To synchronize the whiteflies’ age, adult flies were removed from the cabbage plants, the plants were placed in a bugdorm cage, and kept in a greenhouse for three days. One-to-three-day old adult whiteflies were collected from the cabbage plants and transferred to the BaCV-Luz-infected bean plants for an acquisition accession period (AAP) of 7 days (Figure 3). As a control, a batch of whiteflies was placed for 7 days in healthy bean plants. Next, 25 to 30 potentially viruliferous whiteflies were transferred from the virus-infected source plants to a polyester voile rearing bag previously placed around a trifoliate leaf of 4 healthy beans ‘BRS FC 401 RMD’, 4 healthy soybeans [*Glycine max* (L.) Merr. ‘BR16′], and 4 healthy cowpeas [*Vigna unguiculata* (L.) Walp. ‘BRS Imponente’] plants for an inoculation access period (IAP) of 7 days. Likewise, as controls, non-viruliferous flies were placed in 2 healthy plants of each plant species for 7 days. After this period, the leaves inside the polyester voile bags were detached from plants to avoid nymphal maturation, and plants were sprayed with imidacloprid (32 mg/L) to remove any possible remaining whiteflies [27]. Plants were observed for the development of symptoms, and virus infection was confirmed by RT-PCR, as described above.

### 2.7. BaCV Detection in Plants and Whiteflies

To identify the presence of BaCV-Luz in bean plants and whiteflies, the total RNA was treated with TURBO DNase (Invitrogen, Carlsbad, CA, USA) to eliminate any DNA trace from the RNA preparation as described by Cao, et al. [28]. The cDNA was prepared with Anchored Oligo (dT) 20 primer (Invitrogen, Carlsbad, CA, USA) and SuperScript III Reverse Transcriptase (Invitrogen, Carlsbad, CA, USA) and the PCR reactions were performed with primers specific for all BaCV genes (Table S1). The transcripts for Actin-11 (*act11*) [29] and the small Rubisco subunit (*RbcS*) [30] genes from common bean, and Ribosomal protein L9 (*RpL9*) [31] and Vacuolar ATPase (*v-ATPase*) subunit A [31] genes from whitefly, were used as internal reference controls and to identify possible transcripts ingested by the whiteflies during the feeding in the bean plants (Table S1).

### 2.8. Transmission Electron Microscopy

Small leaf pieces were cut from ‘BRS FC 401 RMD’ bean plants that were experimentally inoculated with whitefly and tested positive for BaCV by RT-PCR. Leaf sections were fixed overnight with Karnovsky modified fixative (2.5% glutaraldehyde and 2% paraformaldehyde in 0.05 M cacodylate buffer, pH 7.2). The samples were post-fixed with 1% osmium tetroxide (in 0.05 M cacodylate buffer) for 1–2 h. Tissues were dehydrated, embedded in low viscosity epoxy Spurr resin (Electron Microscopy Sciences, Hatfield, PA, USA), and sectioned in a Leica UC6 ultramicrotome equipped with a Diatome diamond knife. Ultrathin sections (70–100 nm thick) were transferred onto 300 mesh copper grids, stained with 3% uranyl acetate and Reynold’s lead citrate, and examined in a JEOL JEM 1011 transmission electron microscope. Healthy bean leaves were used as controls. Images were digitally recorded. Whiteflies were reared and collected from BaCV-Luz-infected bean plants, dipped into a NaCl 0.9% solution, and dissected at the thorax region in two parts: head/thorax and abdomen. For each whitefly, either the head/thorax or the abdomen parts were used for BaCV detection by RT-PCR, and the other part was fixed in a cold solution of 2.5% glutaraldehyde and 1.8% sucrose in 0.1 M cacodylate buffer. For the whiteflies that one part of the body was positive for BaCV presence by RT-PCR, the other part was processed to be examined by electron microscopy, as described above for plant tissues. Three pairs of head and abdominal parts of presumably viruliferous whiteflies were examined.

## 3. Results and Discussion

### 3.1. BaCV Is Widely Distributed throughout Common Bean Producing Areas in Brazil

BaCV was identified for the first time in Brazil in 2014 in bean plants collected in Santo Antônio de Goiás, Goiás State (GO) [32]. In June 2016, severe virus-like symptoms were recorded by farmers in Luziânia (GO) (Figure 1). The incidence of these symptoms in the bean plants ranged from 20% to 80%, depending on the area. The bean fields were heavily infested by whiteflies migrating from nearby cotton and tomato fields. Therefore, the first 15 bean plants received from the farmers were screened by PCR or RT-PCR for the presence of the whitefly-borne viruses BGMV, MaYSV, and CPMMV in addition to BRMV, and the new cytorhabdovirus BaCV (Figure S1). None of the plants were infected with MaYSV or BRMV. Conversely, all 15 plants had a mixed infection with BaCV, BGMV, and CPMMV.

**Figure 4:**
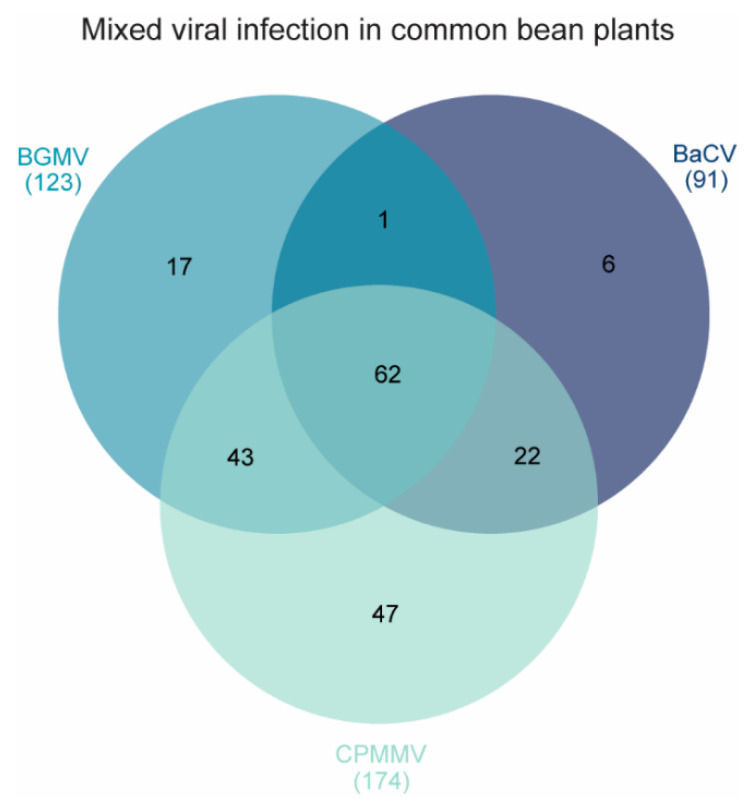
Distribution of viral infections in 219 common bean plants from 2007 to 2018. BaCV was found mostly in mixed infections with BGMV and CPMMV. Only six plants were found singly infected with BaCV.

Given the high infection rate, we further investigated the occurrence of these viruses in newly collected (2016–2018) and archived samples (2007–2016) from Central, Southeast, and Northeastern Brazil, comprising six states and the federal district (Figure 2). In total, 219 plants were analyzed, and 91 (41.55%) were infected by BaCV (Table 1 and Figure 4). Most of the plants analyzed (46.12%) were sampled in Goiás, Central Brazil, one of the top bean-producing States in the country, where the incidence of BaCV reached up to 100%, depending on the area (Table 1). Despite the uneven sampling among regions, we were able to detect BaCV in Southeast and Northeast regions, which are more than 2000 km apart, suggesting that BaCV is widely distributed throughout bean producing areas in the country. However, to have a better view of the prevalence of BaCV in the bean crops in Brazil, further sampling should be conducted in additional bean cultivating areas in the Southeastern, Northeastern, and Southern states.

BaCV was found in a single infection only in six samples of BGMV-resistant ‘BRS FC401 RMD’ plants collected in Riacho Fundo, DF, in 2015. The remaining 85 BaCV positive plants were co-infected with CPMMV (*n* = 22), BGMV (*n* = 1) or with CPMMV and BGMV (*n* = 62) (Table 1, Figure 4). Mixed infections were, therefore, common in these plants. Viral co-infections are very common and, in the field, seem to be the rule rather than the exception, and may result in synergistic effects and stronger symptoms [33]. Moreover, the change in the plant phenotype induced by the co-infection can alter or increase the attraction of vectors and facilitate the transmission of these viruses and enhance epidemics [34]. Unfortunately, singly BaCV-infected samples were identified only in archived samples, and we do not have records of the specific symptoms displayed by these plants. Therefore, it was impossible to establish possible effects of mixed infections on the severe symptoms observed in the field. Importantly, the phenotype of plants in the field is also influenced by other biotic and abiotic factors such as water and temperature stress and infection by other pathogens like bacteria and fungi.

### 3.2. Evolutionary Analysis Indicates Whiteflies as the Potential Vector for BaCV

**Figure 5:**
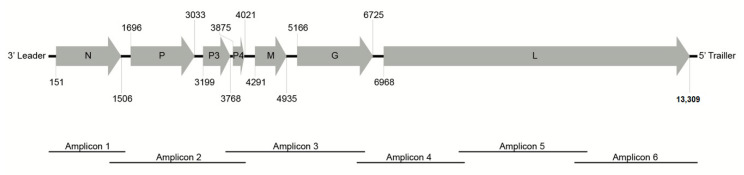
Genome organization of BaCV-Luz. Canonical genes encoding [N] nucleoprotein, [P] phosphoprotein, [M] matrix protein, [G] glycoprotein and [L] RNA-dependent RNA polymerase (RdRp), and non-canonical [P3] and [P4]. Each ORF is represented by a gray arrow with the first and last nucleotide positions depicted. The complete genome (13,467 nts) was recovered by RT-PCR of six overlapping amplicons.

**Figure 6:**
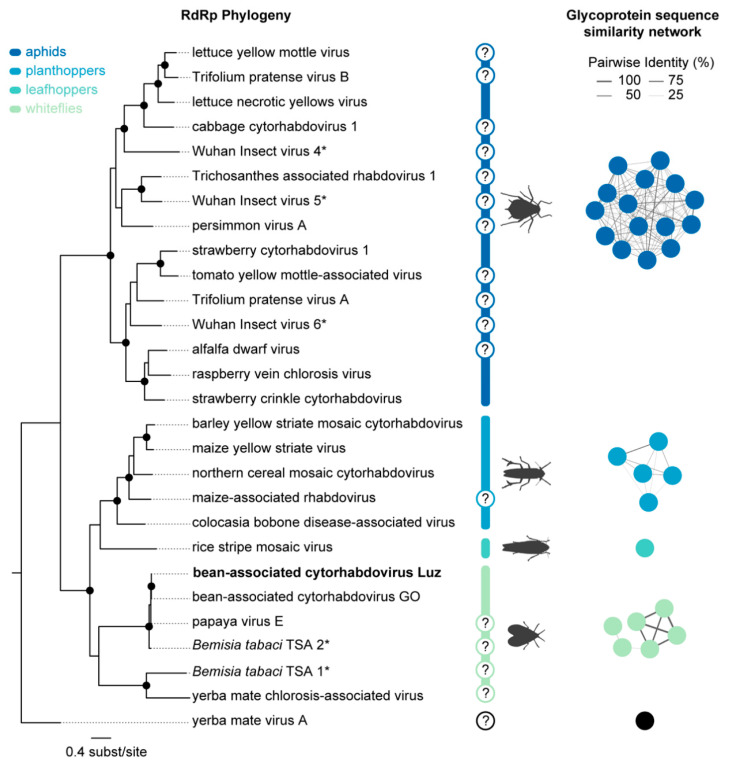
Maximum likelihood phylogenetic tree of cytorhabdovirus RdRP amino acid sequences and sequence similarity network of cytorhabdovirus glycoprotein amino acid sequences encoded by BaCV-Luz, BaCV-GO and other 26 cytorhabdoviruses. In both analyses, four groups were clustered according to their probable vector: aphid, planthopper, leafhopper, and whitefly. Enzyme Function Initiative–Enzyme Similarity Tool (EFI–EST) was used for glycoprotein analysis with an alignment score threshold of 35 and a minimum E-value threshold of 1 × 10^−5^. The network was visualized in Cytoscape v3.7.1. Support values ≥ 90% SH-aLRT are displayed with black circles at nodes. (?) Unknown vector species. (*) Viruses detected by insects metatranscriptome.

The complete BaCV-Luz genome was determined by PCR of six overlapping fragments and was 13,467 nt in length. As expected, the genome presented the seven ORFs originally described in BaCV-GO (N, P, P3, P4, M, G, and L), flanked by two non-transcribed leader and trailer regions (Figure 5). As shown in Table S3, the complete genome of BaCV-Luz shares 99.8% and 96.3% identity with BaCV-GO and PpVE, respectively. To further investigate the phylogenetic relationship of cytorhabdoviruses, we aligned 28 RdRP amino acid sequences (2374 aa in length, including gaps) from reference cytorhabdoviruses available in Genbank, including two sequences derived from transcriptomes of *B. tabaci* (Table S2). The phylogenetic analysis shows that cytorhabdoviruses cluster in monophyletic groups according to its potential vector: aphid [35,36,37,38,39], planthopper [40,41,42,43,44,45], leafhopper [46], whitefly, and an undescribed vector (Figure 6). As previously suggested [7,47,48], BaCV isolates and PpVE were closely related to *B. tabaci* TSA 2 (AKC57270.1), confirming that these viruses belong to the species *Papaya cytorhabdovirus*. Moreover, yerba mate chlorosis-associated virus clustered with *B. tabaci* TSA 1 (AKC57275.1), as a sister group of papaya cytorhabdovirus strains. Furthermore, assuming that the viral surface envelope glycoprotein G interacts directly with receptors in the vector cells, a sequence similarity network (EFI–EST webserver) was generated using the glycoprotein amino acid sequences encoded by the 28 cytorhabdoviruses. Interestingly, the analyses show three distinct clusters (aphids, planthopper, and whiteflies) and two singletons (leafhopper and an undescribed vector) with a high degree of interconnectivity.

These results, together with the detection of BaCV in concomitant infection with the whitefly vectored BGMV and CPMMV in all plants (Figure 4), and with the presence of whiteflies adults, nymphs, and eggs in these plants (Figure 1), prompted us to postulate that whiteflies also vector BaCV.

### 3.3. B. tabaci MEAM1 Transmit BaCV-Luz to Common Beans, Cowpea, and Soybean

The known vectors of cytorhabdoviruses are insects belonging to the families Aphididae (aphids), Delphacidae (planthoppers), and Cicadellidae (leafhoppers). In general, when the vectors are known, there is a highly specific relationship, and only one (or a few related) types or species of a vector are capable of transmitting a given virus. Thus, it is possible to establish a strong correlation between viral detection and the presence of its vector in the field [3,49].

**Figure 7:**
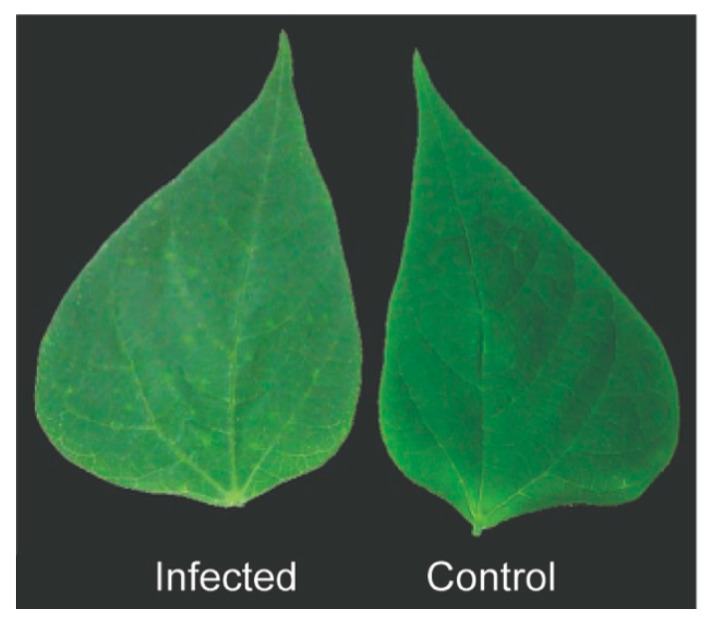
Common bean ‘BRS FC 401 RMD’ leaves from whitefly mediated BaCV and CPMMV transmission. Mild mottling and chlorotic spots in a leaf of an infected plant at ~50 days after inoculation and the leaf of a non-infected control plant.

The field in Luziânia, GO, where the bean plants were initially sampled, was densely infested by whiteflies. The genotyping of these whiteflies by PCR-RFLP confirmed their identity as *B. tabaci* MEAM1, the prevalent whitefly species in Central Brazil [50]. To evaluate whether whiteflies also transmit BaCV, we used a BaCV (plus BGMV and CPMMV) field-collected infected bean plant as inoculum source and the flies carried by this plant in a free choice transmission assay (Figure 3). After a 14 day exposition time to the whitefly feeding, 100% (*n* = 48) of the bean plants ‘Pérola’, ‘Jalo’, and ‘BRS FC 401 RMD’ tested positive for the presence of BaCV RNA by RT-PCR. Whitefly adults collected at the same time as bean leaves were also positive for BaCV and CPMMV (Figure S2). All the plants also contained CPMMV, and the susceptible cultivars were positive for BGMV, suggesting that all three viruses were simultaneously transmitted by whiteflies. The mild mottling symptoms detected in ‘BRS FC 401 RMD’ plants ~50 days after introduction into the cage (Figure 7) resemble those described for CPMMV infection [51]. These results indicate that BaCV could be transmitted at high rates to three different bean cultivars by whiteflies *B. tabaci* MEAM1. The BaCV-Luz isolate was maintained by whitefly-mediated periodical transmission to healthy bean plants for 18 months. Altogether, during this period, 83 plants were exposed to potentially viruliferous flies in the cage, and 72 became infected by BaCV, an overall transmission rate of ~87%.

A second experiment was conducted to confirm the capacity of whiteflies to transmit BaCV to common bean as well as soybean and cowpea (Figure 3). Using 25 to 30 adult *B. tabaci* MEAM1 per plant and both AAP and IAP of 7 days, BaCV transmission was achieved in 75% of the common beans, 50% of cowpeas, and 25% of the soybean plants (Table 2). All control plants exposed to non-viruliferous insects tested negative for BaCV (and CPMMV). With these results, it was also possible to experimentally extend the host range of BaCV to cowpea and soybean (Table 2). The second experiment showed a transmission efficiency rate lower than the pilot test, in which transmission occurred to 100% of the plants. The reduction in the efficiency of BaCV transmission may be related to the IAP, which was shorter than in the first experiment. Besides, the number of adult insects feeding on each plant was limited to a maximum of 30, while in the first test, the whiteflies were free to feed so that each plant may have received a larger number of viral particles.

Furthermore, the whiteflies’ ages were not synchronized in the initial trials, and the insect stage of the life cycle may influence the efficiency of BaCV transmission. Soybean is an economically important cash crop in Brazil and is also susceptible to CPMMV [52] and BGMV [53]. Cultivation cycle of soybean and common bean overlap in many areas of Brazil. In these areas, soybeans may act as an inoculum source of these three whitefly-borne viruses to bean crop hasting multiple virus epidemics. Our future investigation should focus on the study of field infection of soybean plants by BaCV. *B. tabaci* is a complex containing approximately 40 cryptic species with similar morphology but differing in the genetics, behavior, efficiency as a virus vector, and in the colonization by endosymbionts [50,54,55]. *B. tabaci* has a large number of hosts, more than 500 plant species, cultivated or not, in tropical and subtropical regions. *B. tabaci* is considered a super vector since it transmits over 300 plant viruses including begomoviruses (family *Geminiviridae*), criniviruses (family *Closteroviridae*), torradoviruses (family *Secoviridae*), ipomoviruses (family *Potyviridae*), and the carlaviruses CPMMV and melon yellowing-associated virus (MYaV) (family *Betaflexiviridae* [56,57]). Recently, two poleroviruses (family *Luteoviridae*) were also shown to be transmitted by *B. tabaci* [58,59].

Our transmission results demonstrate that whiteflies, in this case the species *B. tabaci* MEAM1, are vectors of the cytorhabdovirus BaCV in Brazil, highlighting the importance of whiteflies as plant virus vectors and emphasizing their designation as super vectors [56]. Moreover, our results expand the whiteflies’ attributes as vectors, including the cytorhabdovirus group, to the list of viruses they can transmit.

Despite whiteflies being successful plant virus vectors, the transmission efficiency may vary depending on the virus, virus isolate, host plant, whitefly species and biology, and virus and whitefly population’s geographical origin [57]. In our study, *B. tabaci* MEAM1 was very efficient in transmitting BaCV, especially to bean plants. *B. tabaci* MEAM1 predominates as a vector of various plant viruses in Brazil [50]. However, other whitefly species such as *B. tabaci* Mediterranean (MED) and *B. tabaci* New World (NW) are also present in more restricted areas of Southeastern and Southern States. Whether whiteflies MED and NW can transmit BaCV to beans or other crops remains to be investigated.

It is also necessary to investigate if *B. tabaci* MEAM1 is the vector of PpVE to papaya plants in Ecuador and if this virus infects papayas in Brazil. Cornejo-Franco, et al. [60] mention that *B. tabaci* is a major pest of papayas in Ecuador and is the vector of papaya virus Q (an umbra-like virus). Thus, this whitefly species may also be the vector PpVE to papayas. We have tested a limited number of papaya plants (*n* = 27) collected in the Distrito Federal and the State of Espirito Santo for the presence of BaCV with negative results. Nine whitefly species, including *B. tabaci* MEAM1, were already identified on papaya trees in Brazil, but *Trialeurodes variabilis* is the primary species associated with this fruit crop, and *B. tabaci* MEAM1 has a limited occurrence in papayas in the country [61]. Considering that BaCV and PpVE are frequently detected in mixed infections, it is important to investigate if this group of viruses is transmitted alone by *B*. *tabaci* MEAM1 or if a helper virus is required.

### 3.4. BaCV-Luz Detection in Plants and Whiteflies

Depending on the taxonomic group they belong to, viruses are transmitted by their whitefly vectors by different transmission modes [57]. The carlavirus CPMMV is reported to be stylet borne and transmitted in a nonpersistent mode [52]. While criniviruses, torradoviruses, and ipomoviruses are semipersistent viruses and foregut borne, the mode of transmission of poleroviruses by *B. tabaci* is still unknown [56,57]. Begomoviruses are transmitted by different species in the *B. tabaci* complex in a persistent circulative manner. However, at least for one begomovirus, tomato yellow leaf curl virus (TYLCV), there is evidence that it replicates in the whitefly [62,63,64,65] and that replication takes place mainly in the salivary glands [66].

Plant rhabdoviruses are transmitted by their arthropod vectors in a persistent, circulative-propagative manner. After the acquisition, viruliferous insects transmit plant rhabdoviruses for their entire lives. In plants, rhabdoviruses infect, replicate, and accumulate in a variety of tissues, including leaf epidermis and mesophyll, phloem tissues, and roots [5,67]. In their insect vectors, plant rhabdoviruses infect gut cells, muscle cells, nervous tissue, hemocytes, tracheae, and salivary glands [3,5,28].

**Figure 8:**
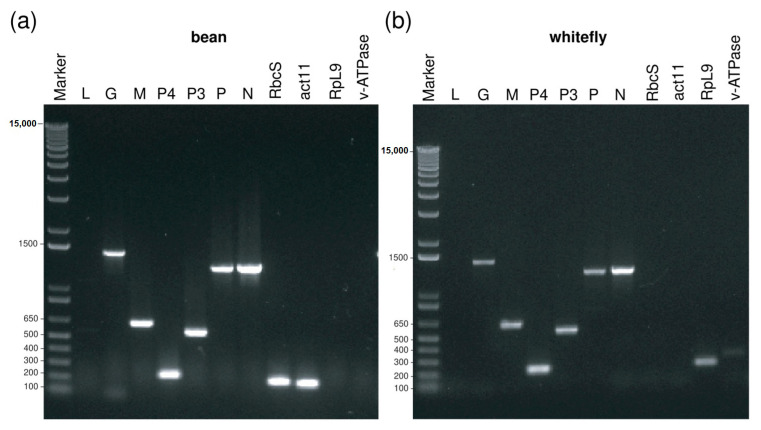
BaCV detection in common bean leaf and whiteflies (*B. tabaci* MEAM1). BaCV RNA corresponding to G, M, P4, P3, P, N genes were detected by PCR using specific primers. Internal controls include the plant *RbcS* and *act11* and the whitefly *RpL9* and *v-ATPase*. (**a**) RT-PCR products obtained from RNA extracted from common bean. (**b**) RT-PCR products obtained from RNA extracted from viruliferous whiteflies.

BaCV RNA corresponding to N, P, P3, P4, M, G genes were amplified from BaCV-infected bean leaves and potentially viruliferous whiteflies collected in infected plants. Fragments with sizes according to their predicted ORFs were amplified (Figure 8a,b), except for L, probably because of its large size. Amplicons corresponding to *act11* and *RbcS* were only amplified from bean cDNA, and *v-ATPase* and *RpL9* from whiteflies (Figure 8a,b).

**Figure 9:**
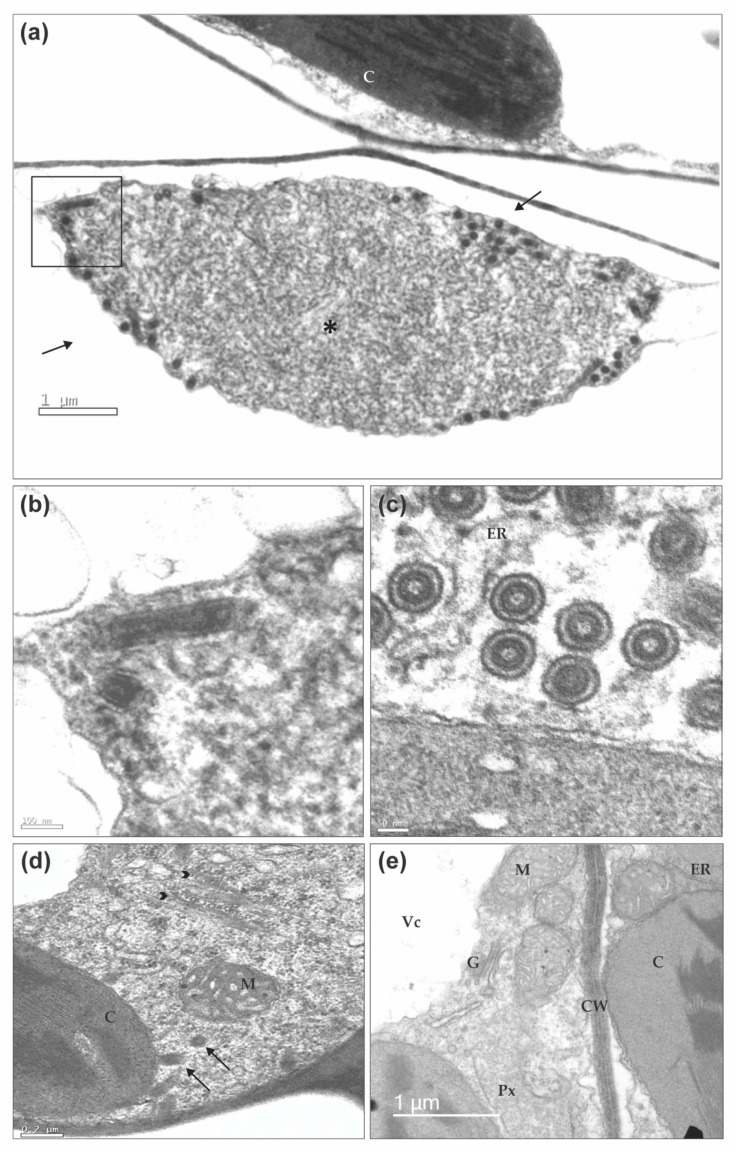
Transmission electron micrographs of bean leaf infected by BaCV. (**a**) Overview of a viroplasm formed by coiled filamentous material (*) in the cytoplasm of a parenchymal cell. Typical rhabdovirus particles are present in the periphery of the viroplasm (arrows). (**b**) Details of the marked square with longitudinally-sectioned particles are depicted. (**c**) Cross-sections of BaCV particles show the internal and cylindrical nucleoprotein core, and the outer viral membrane, and also that virions are within a cavity of the endoplasmic reticulum. (**d**) Spongy parenchymal cell dually infected by BaCV and CPMMV. Brush-like aggregates of CPMMV particles (arrowheads) and BaCV in longitudinal and cross-sections (arrows) are visible. (**e**) Palisade parenchyma cells from a healthy bean plant. Chloroplast (C), endoplasmic reticulum (ER), Golgi complex (G), mitochondrion (M), peroxisome (Px), vacuole (Vc), and cell wall (CW).

We have used transmission electron microscopy (TEM) to visualize BaCV accumulation in bean and whitefly tissues. Electron microscopic examination of BaCV-infected bean leaves revealed bacilliform particles typical of rhabdoviruses in parenchymal cells. Longitudinal and cross-sectioned particles were located in the cell cytoplasm, commonly at the periphery of an electron-lucent mass of coiled filamentous material, believed to be the viroplasm, where the virus replicates (Figure 9a–d) [5,68]. The BaCV bacilliform particles seemed rather scarce in the observed bean tissues. Presumed cytorhabdovirus virions were found in only one of the four examined leaf samples, though all plant samples tested positive for BaCV by RT-PCR.

By contrast, feather-like aggregates of CPMMV particles were readily recognized in all examined bean tissue samples. These in situ observations of BaCV and CPMMV virions presence in dually infected bean leaf corroborate the previous HTS sequencing study in which 8.2% of the total sequence reads from multiple virus-infected bean leaves corresponded to CPMMV whereas only 0.01% mapped to BaCV [6]. In some cases, BaCV and CPMMV virions could be identified infecting the same cell (Figure 9d). Unfortunately, sections of three pairs of head/thorax or abdominal parts of whiteflies that fed in BaCV-infected beans did not yield evidence of BaCV particles or viroplasm in the observed tissues. The insects were tested before fixation, and only head/thorax corresponding to insects that the abdomen tested positive for BaCV or vice-versa was examined. Therefore, albeit BaCV RNA could be detected in the insects by RT-PCR, particles were not localized in any of the tissues examined.

The spatial and temporal distribution of BaCV within the whitefly body could have hindered the localization of particles by TEM. Moreover, plant rhabdoviruses appear to replicate and accumulate at lower levels in insect cells when compared to plant cells [3,6,69]. BaCV seems to accumulate at low levels, even in bean plants. Therefore, assessment of dissected organs such as midguts or salivary glands could facilitate the visualization of BaCV in whiteflies either by TEM or by confocal microscopy using immunofluorescence or in situ hybridization. The occurrence of BaCV vRNA, cRNA, and mRNA in whiteflies will be investigated in future studies.

## 4. Conclusions

Our results show that BaCV is efficiently transmitted, in experimental conditions, by *B. tabaci* MEAM1 to three bean cultivars grown in Brazil, and with lower efficiency to cowpea and soybean. It remains to be determined whether BaCV replicates in whiteflies, as observed for other plant-infecting rhabdoviruses in their arthropod vectors. BaCV could not be singly isolated for pathogenicity tests, identification of the induced symptoms, and the transmission assay. Moreover, BaCV was detected in five out of seven Brazilian states evaluated. Besides BaCV in Brazil (this study, [6]) and PpVE in Ecuador [7], similar virus sequences were recorded from whiteflies samples in India [70], beans from Tanzania [71], and citrus, passion fruit, and paper bush in China [72], implying that other isolates/strains of BaCV/PpVE or related rhabdoviruses that are also transmitted by whiteflies might exist in other continents.

## Figures and Tables

**Table 1:** Detection of BaCV, CPMMV, and BGMV in common bean samples collected in Brazil by RT-PCR and PCR.

State/Total Number of Samples	City	Sampled Areas	Year	Virus Incidence (Infected/Tested)		
				BaCV	CPMMV	BGMV
Bahia (BA) (*n* = 17)	Cruz das Almas	1	2015	2/8	8/8	4/8
	Morro do Chapéu	1	2015	0/4	3/4	0/4
	Piritiba	1	2015	0/3	3/3	0/3
	Antônio Gonçalves	1	2015	0/2	2/2	0/2
Total in Bahia				2/17	16/17	4/17
Alagoas (AL) (n = 5)	Arapiraca	1	2018	0/5	4/5	1/5
Total in Alagoas				0/5	4/5	1/5
Minas Gerais (MG) (n = 9)	Bonfinópolis de Minas	1	2016	0/3	3/3	0/3
	Paracatu	1	2016	1/1	1/1	1/1
	Três Pontas	1	2018	0/5	1/5	2/5
Total in Minas Gerais				1/9	5/9	3/9
São Paulo (SP) (n = 1)	Palmital	1	2015	1/1	1/1	1/1
Total in São Paulo				1/1	1/1	1/1
Goiás (GO) (n = 101)	Luziânia	1	2016	15/15	15/15	15/15
		2	2016	7/7	7/7	7/7
		3	2016	3/4	4/4	4/4
		4	2016	4/4	4/4	4/4
	Cristalina	1	2016	10/14	14/14	14/14
		2	2016	0/14	12/14	13/14
		3	2016	11/11	10/11	11/11
		4	2016	4/4	4/4	4/4
	Santo Antônio de Goiás	1	2016	0/4	4/4	4/4
		2	2016	2/17	17/17	8/8
		1	2018	0/5	4/5	4/5
	Urutaí	1	2018	0/1	0/1	0/1
	Araçu	1	2018	0/1	1/1	1/1
Total in Goiás				56/101	96/101	89/92
Distrito Federal (DF) (n = 84)	Brasília	1	2017	8/30	19/30	14/30
		1	2007	0/2	0/2	2/2
		1	2012	0/11	1/11	7/11
		1	2015	8/12	6/12	-
		2	2015	15/29	25/29	-
Total in Distrito Federal				31/84	51/84	23/43
Mato Grosso (MT) (n = 2)	Sorriso	1	2018	0/2	1/2	2/2
Total in Mato Grosso				0/2	1/2	2/2
TOTAL				91/219	174/219	123/169

**Table 2:** BaCV transmission by *B. tabaci* MEAM1.

Plant	Cultivar	Positive/Total	Transmission Rate (%)
*P. vulgaris* (common bean)	‘BRSFC 401 RMD’	3/4	75
*V. unguiculata* (cowpea)	‘BRS Imponente’	2/4	50
*G. max* (soybean)	‘BR16′	1/4	25

## Supplementary Materials

The following are available online at https://www.mdpi.com/1999-4915/12/9/1028/s1, Figure S1: RT-PCR and PCR detection of bean-infecting viruses, Figure S2: RT-PCR detection of BaCV and CPMMV, Table S1: Primers used in this study, Table S2: Accession number of cytorhabdoviruses sequences in GenBank and their known vectors, Table S3: Nucleotide and amino acid sequence identities (%) of BaCV-Luz ORFs compared with PpEV strains.
