# Supplementary material for: Transmission of the Bean-Associated Cytorhabdovirus by the Whitefly Bemisia tabaci MEAM1
Source: Viruses. 2020 Sep 15;12(9):1028. doi: 10.3390/v12091028 (PMC7551397; doi:10.3390/v12091028)
Supplement: Supplementary file 1 [file viruses-12-01028-s001.zip › Pinheiro-Lima_etal_2020_SupplementaryFigures.docx]

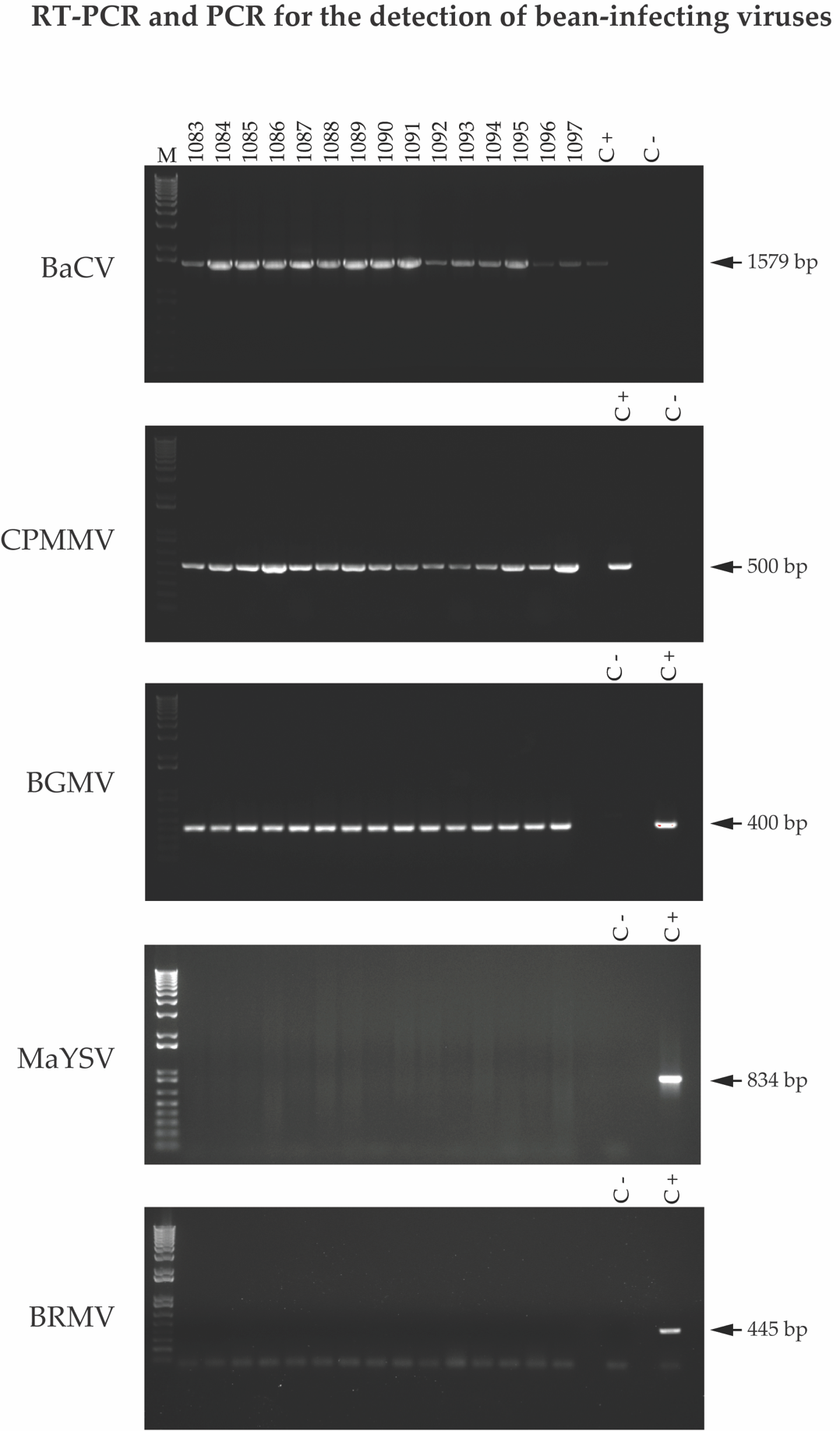


**Supplementary Figure 1**. RT-PCR and PCR detection of bean-infecting viruses: BaCV, CPMMV, BGMV, MaYSV, BRMV. The expected product size is shown. M: 1 Kb Plus DNA Ladder (Thermo Fisher Scientific).


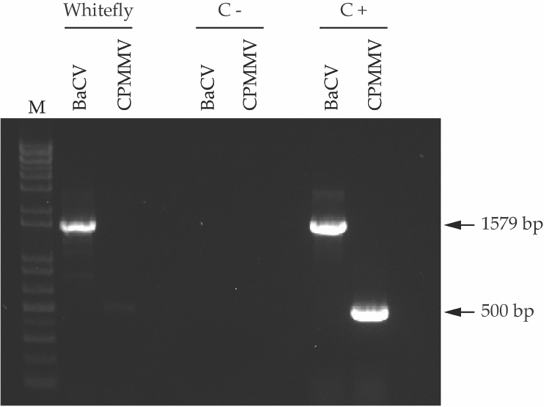


**Supplementary Figure 2**. RT-PCR detection of BaCV and CPMMV. The expected product size is shown. M: 1 Kb Plus DNA Ladder (Thermo Fisher Scientific).
