# Supplementary material for: Transmission of the Bean-Associated Cytorhabdovirus by the Whitefly Bemisia tabaci MEAM1
Source: Viruses. 2020 Sep 15;12(9):1028. doi: 10.3390/v12091028 (PMC7551397; doi:10.3390/v12091028)
Supplement: Supplementary file 1 [file viruses-12-01028-s001.zip › Pinheiro-Lima_etal_2020_SupplementaryTables.docx]

**Table S1.** Primers used in this study.

| **Target** | **Primer name^1^** | **Primer sequence 5’- 3’** | **Amplicon size (bp)** | **Tm (**°C) | **Reference** |
| --- | --- | --- | --- | --- | --- |
| BaCV | BaCV_1F | GTTCAAAACTTTATAACCGCAGGAG | 1579 | 61°C | This study |
|  | BaCV_1579R | CGCAACAGATTAAACAGGAAAT |  |  |  |
| CPMMV | CPMMV_4000F | AACTTGGCCTTAGTGAACTCTACA | 500 | 61°C | [15] |
|  | CPMMV_4500R | ATTAGCTCTGTGCCTGGGGT |  |  |  |
| BRMV | BRMV1_76F | CTTGACTTGGTTGAAATACACCT | 445 | 62°C | [6] |
|  | BRMV1_521_R | GCCCTCAGCTTGACTAGGCCC |  |  |  |
| BGMV | BGMV_HPXHO | CCTCGAGATAGTGCGGTGCGA | 400 | 65°C | [16] |
|  | BGMV_HPKPN | AGGTACCATGCGGCATCCGAAGC |  |  |  |
| MaYSV2^2^ | MaYSV-249F | GGTTTCCCGCAATAACAA | 834 | 55°C | This study |
|  | MaYSV-1083R | CATGTTGTACTTAGAACGAA |  |  |  |
| N gene | BaCV_N_F | ATGCCGAAAGCGTACAAGGAC | 1356 | 60°C | This study |
|  | BaCV_N_R | TCAAGGCTTGTAGATCCTCTTGC |  |  |  |
| P gene | BaCV_P_F_2 | ATGTCTATTGAGGAGATGG | 1338 | 55°C | This study |
|  | BaCV_P_R | TTAATAGTATGACTTGATATTCTTC |  |  |  |
| P3 gene | BaCV_P3_F | ATGGAGAAGAGCAACTCAGATA | 570 | 58°C | This study |
|  | BaCV_P3_R | TTATTCAGTGGGAATCAAGGAGA |  |  |  |
| P4 gene | BaCV_P4_F | ATGGAAGGACAATGCAGG | 237 | 55°C | This study |
|  | BaCV_P4_R | TCACTCATCCCCACTAGG |  |  |  |
| M gene | BaCV_M_F | ATGTCAATCCTAAAGTATATCTC | 645 | 55°C | This study |
|  | BaCV_M_R | TCATTTAACAGACTTAAACAGAG |  |  |  |
| G gene | BaCV_G_F | ATGTCTGGGTACGCTATTGC | 1560 | 60°C | This study |
|  | BaCV_G_R | CTACGCAAAGATGGGGAACG |  |  |  |
| L gene | BaCV_L_F | ATGTCTTTCTTCTTTGAGGAA | 6342 | 51°C | This study |
|  | BaCV_L_R | TCAGTCAGCAATGAAAGC |  |  |  |
| *RbcS* | CG03_Pv_s_rubiscoF | ttggagcatggtttcgtgta | 180 | 55°C | [30] |
|  | CG03_Pv_s_rubiscoR | atgcactgcacttgacgaac |  |  |  |
| *Act11* | qAct11_F | TGCATACGTTGGTGATGAGG | 150 | 58°C | [29] |
|  | qAct11_R | AGCCTTGGGGTTAAGAGGAG |  |  |  |
| Whitefly *RpL9* | qWF_RPL9_F | AGGTAGACATGTTCCGTGGTGT | 250 | 55°C | [31] |
|  | qWF_RPL9_R | CACAACTTTGCATGACGTGTGG |  |  |  |
| Whitefly v-ATPase | qWF_ATPase_F | TTCCGGACGTTTGGCAGAGA | 300 | 55°C | [31] |
|  | qWF_ATPase_R | ACCGCGCCAACAATACTCACA |  |  |  |
| cDNA | M10Pa-clT50VN | AAGCAGTGTTATCAACGCAGATTAATTAAT_50_VN | - | - | [12] |
| cDNA | Anchored Oligo(dT)20 | (T)20VN | - | - | Invitrogen |
| RACE 3’ First PCR | M10 | AAGCAGTGTTATCAACGCAGA | 580 | 61°C | [6,12] |
|  | BaCV_12881F | GGAGGATCATGAGAGACTGAGGC |  |  |  |
| RACE 3’ Second PCR | M10 | AAGCAGTGTTATCAACGCAGA | 300 | 61°C | [6,12] |
|  | BaCV_13169F | GGTGACCTTCCAGTACCTTCCTC |  |  |  |
| RACE 5’ First PCR | AAP | GGCCACGCGTCGACTAGTACGGGIIGGGIIGGGIIG | 600 | 61°C | [6,13] |
|  | BaCV_546_R | TTCACTCTCCTCAGACTCCTTGC |  |  |  |
| RACE 5’ Second PCR | AUAP | GGCCACGCGTCGACTAGTAC | 400 | 61°C | [6,13] |
|  | BaCV_321_R | TGTACATCCCATACCGCTCCAG |  |  |  |
| Amplicon 1 | BaCV_1F | GTTCAAAACTTTATAACCGCAGGAG | 1579 | 60°C | This study |
|  | BaCV_1579R | CGCAACAGATTAAACAGGAAAT |  |  |  |
| Amplicon 2 | BaCV_1248F | ACAGAGGAGACTGGGGATCC | 2813 | 60°C | This study |
|  | BaCV_4061R | ATGCATGTTACTTTCTACACAC |  |  |  |
| Amplicon 3 | BaCV_3645F | GGACGGCGACTATCTGACAG | 2874 | 60°C | This study |
|  | BaCV_6519R | CGACGAGCTATGCGACTTCT |  |  |  |
| Amplicon 4 | BaCV_6369F | CTCGGGGGATGTCTCGAAATC | 2242 | 60°C | This study |
|  | BaCV_8611R | TCCGTTACGACAATATAAGCCC |  |  |  |
| Amplicon 5 | BaCV_8495F | GTTGCCAAAGAAGTGTCGGAT | 2654 | 60°C | This study |
|  | BaCV_11149R | CCGGAATAAGAGAATTTGGACGC |  |  |  |
| Amplicon 6 | BaCV_10947F | CCCGTACCTCCTTCCTCCAT | 2522 | 60°C | This study |
|  | BaCV_13469R_GA | GAGATGCCATGCCGACCCGCGAACGAAAATATCGAAA |  |  |  |
| Whitefly mtCOI | COI-Fw | TTGATTTTTTGGTCATCCAGAAGT | 880 | 45°C | [17] |
|  | COI-Rv | TCCAATGCACTAATCTGCCATATTA |  |  |  |

^1^ F: Forward, R: Reverse. ^2^Sequence accession number: JN419005.1.

**Table S2.** Accession number of cytorhabdoviruses sequences in GenBank and their known vectors or insect hosts.

| **Acronym** | **Virus** | **Accession number** | **Vector or insect Host (Family)** | **Reference** |
| --- | --- | --- | --- | --- |
| ADV | alfalfa dwarf virus | KP205452.2 | Unknown |  |
| BYSMV | barley yellow striate mosaic virus | KM213865.1 | *Laodelphax striatellus* (Delphacidae) | [40] |
| BaCV-GO | bean-associated cytorhabdovirus | MK202584.1 |  |  |
| BaCV-Luz | bean-associated cytorhabdovirus-Luz | MT811775.1 | *B. tabaci* MEAM1  (Aleyrodidae) | This study |
|  | *B. tabaci* TSA 1 | KJ994265.1/GAUC01021749.1 | *B. tabaci* (Aleyrodidae)* |  |
|  | *B. tabaci* TSA 2 | KJ994260.1/KJ994255.1 | *B. tabaci* (Aleyrodidae)*** |  |
| CCyV-1 | cabbage cytorhabdovirus 1 | KY810772.1 | Unknown |  |
| CBDaV | colocasia bobone disease-associated virus | KT381973.1 | *Tarophagus proserpina* (Delphacidae) | [41] |
| LNYV | lettuce necrotic yellows virus | AJ867584.2 | *Hyperomyzus lactucae* (Aphididae) | [35] |
| LYMoV | lettuce yellow mottle virus | EF687738.1 | Unknown |  |
| MaCV | maize associated rhabdovirus | KY965147.1 | Unknown |  |
| MYSV | maize yellow striate virus | KY884672.1 | *Peregrinus maidis* and *Delphacodes kuscheli* (Delphacidae) | [42,43] |
| NCMV | northern cereal mosaic virus | AB030277.1 | *Laodelphax striatellus*,  *Unkanodes albifascia*, *U. sapporona* and  *Muellerianella fairmairei*  (Delphacidae) | [44,45] |
| PpVE | papaya cytorhabdovirus | MH282832.1 | Unknown |  |
| PeVA | persimmon virus A | AB735628.2 | Unknown |  |
| RVCV | raspberry vein chlorosis virus | MK240091.1 | *Aphis idaei* (Aphididae) | [36] |
| RSMV | rice stripe mosaic virus | KX525586.2 | *Recilia dorsalis* and  *Nephotettix virescens* (Cicadellidae) | [46] |
| SCV | strawberry crinkle cytorhabdovirus | MH129615.1 | *Chaetosiphon fragaefolii* and *C. jacobi.* (Aphididae) | [37] |
| StrV-1 | strawberry cytorhabdovirus 1 | MK211270.1 | *Aphis fabae*,  *Acyrthosiphon malvae* and  *A. ruborum*  (Aphididae) | [38] |
| TYMaV | tomato yellow mottle-associated virus | KY075646.1 | Unknown |  |
| TrARV1 | Trichosanthes associated rhabdovirus 1 | BK011194.1 | Unknown |  |
| TPVA | Trifolium pratense virus A | MH982250.1 | Unknown |  |
| TPVB | Trifolium pratense virus B | MH982249.1 | Unknown |  |
| WuIV-4 | Wuhan Insect virus 4 | KM817650.1 | *Hyalopterus pruni* (Aphididae)* | [39] |
| WuIV-5 | Wuhan Insect virus 5 | KM817651.1 | *Hyalopterus pruni* (Aphididae)* | [39] |
| WuIV-6 | Wuhan Insect virus 6 | KM817652.1 | *Hyalopterus pruni* (Aphididae)* | [39] |
| YmCaV | yerba mate chlorosis-associated virus | KY366322.2 | Unknown |  |
| YmVA | yerba mate virus A | MN781667.1 | Unknown |  |

^*^Viruses detected by insects metatranscriptome.

**Table S3.** Nucleotide and amino acid sequence identities (%) of BaCV-Luz ORFs compared with PpEV strains.

|  | Genome | N | | P | | P3 | | P4 | | M | | G | | L | |
| --- | --- | --- | --- | --- | --- | --- | --- | --- | --- | --- | --- | --- | --- | --- | --- |
|  | nt | nt | aa | nt | aa | nt | aa | nt | aa | nt | aa | nt | aa | nt | aa |
| *B.tabaci* TSA 2 | 77.1 | 82.6 | 92.3 | 78 | 82.9 | 81.1 | 88.9 | 73.3 | 71.8 | 81.2 | 93.5 | 76.9 | 86.1 | 80 | 93.2 |
| BaCV-GO | 99.8 | 99.8 | 99.4 | 99.6 | 99.3 | 99.7 | 99.5 | 100 | 100 | 99.8 | 99.5 | 99.4 | 98.7 | 100 | 100 |
| PpVE | 96.3 | 96.6 | 96.3 | 96.6 | 94.2 | 95.9 | 96.8 | 93.7 | 87.2 | 96.4 | 98.1 | 96.7 | 96.5 | 97.3 | 97.8 |
